# RGO and Three-Dimensional Graphene Networks Co-modified TIMs with High Performances

**DOI:** 10.1186/s11671-017-2298-z

**Published:** 2017-09-06

**Authors:** Tang Bo, Wang Zhengwei, Weiqiu Huang, Li Sen, Ma Tingting, Yu Haogang, Li Xufei

**Affiliations:** School of Petroleum Engineering, Changzhou University, Changzhou City, 213016 China

**Keywords:** Thermal Interface Materials, Graphene, Thermal Boundary Resistance

## Abstract

With the development of microelectronic devices, the insufficient heat dissipation ability becomes one of the major bottlenecks for further miniaturization. Although graphene-assisted epoxy resin (ER) display promising potential to enhance the thermal performances, some limitations of the reduced graphene oxide (RGO) nanosheets and three-dimensional graphene networks (3DGNs) hinder the further improvement of the resulting thermal interface materials (TIMs). In this study, both the RGO nanosheets and 3DGNs are adopted as co-modifiers to improve the thermal conductivity of the ER. The 3DGNs provide a fast transport network for phonon, while the presence of RGO nanosheets enhances the heat transport at the interface between the graphene basal plane and the ER. The synergy of these two modifiers is achieved by selecting a proper proportion and an optimized reduction degree of the RGO nanosheets. Moreover, both the high stability of the thermal conductivity and well mechanical properties of the resulting TIM indicate the potential application prospect in the practical field.

**Electronic supplementary material:**

The online version of this article (10.1186/s11671-017-2298-z) contains supplementary material, which is available to authorized users.

## Background

Graphene-assisted thermal interface materials (TIMs) have attracted increasing attention because of their high thermal and mechanical performances [1–5]. Kim et al. reported that the resulting thermal conductivity is 1400% higher than the pristine epoxy resin (ER), and Joen’s group found that a 10 wt% additional graphene filler will bring about a high thermal conductivity (~ 2 W/mK) [3, 4]. However, considering the theoretical thermal conductivity of this unique material is as high as 5000 W/mK [6], the reported results are far from satisfactory. Although graphene is expected to act as the fast transport channel for phonon in the TIMs during the thermal transport process, the nano-scaled RGO sheets lack a continuous structure to form the transport network. Moreover, overmuch interfaces of the RGO nanosheets lead to a high total thermal boundary resistance (Kapitza scattering), which results in a strong phonon scattering [7]. At last, the high defect density of the RGO nanosheets due to the violent oxidation-reduction processes also brings about an extra thermal resistance source (shortening the mean free path of phonon, Umklapp scattering) [8].

In order to give full play to the high thermal conductivity of the adopted graphene, high-quality three-dimensional graphene networks (3DGNs) prepared by chemical vapor deposition method have been adopted to hybridize with ER by our group [7]. The better thermal and mechanical properties of the 3DGNs-ER (compared with that of the RGO-based sample) manifest the fatal significance of the low defect density and the continuous construction of the employed graphene [9]. On the other hand, originating from the absence of surface functional groups of the 3DGNs, a bottleneck, a bed contact between the 3DGNs and ER (a poor wettability of the 3DGNs), is revealed with the ongoing study. Based on our recent report, a moderated amount of surface defects of the 3DGNs can play as a positive role to improve the contact between the graphene basal plane and matrix [10, 11]. However, some tedious adjustment processes including a precise CH_4_ flow and a strict cooling rate of the substrate are needed during the CVD procedure [12]. Therefore, an idea on combining the RGO nanosheets and 3DGNs to utilize their advantages is naturally presented.

In this study, the RGO nanosheets and 3DGNs are adopted as fillers to enhance the thermal performances of the resulting ER. The specific functions of these two modifiers are discussed and proven. On the one hand, the 3DGNs provide a fast transport network, increasing the average mean path of phonons. On the other hand, the RGO nanosheets on the 3DGN surface improve the contact at the interface of the graphene basal plane and ER remarkably, which depresses the interface scattering of phonons. The further improvement of the resulting thermal performance resulting from the synergy of the RGO nanosheets and 3DGNs indicates that utilizing graphene with an optimizing manner is a useful strategy to prepare the high-performance TIMs.

## Methods

### Materials

Nickel foam with 300 gm^−2^ in areal density and 12 mm in thickness was purchased from Haobo Co., Ltd. (Shenzhen, China) and used as a template to fabricate the 3DGNs. Ethanol, HCl, FeCl_3_, and poly(methyl methacrylate) (PMMA, average molecular mass 996,000, 4% in ethyl lactate) were obtained commercially from the Beijing chemical reagent plant (Beijing, China). Ethyl lactate, natural graphite, poly(methyl methacrylate), and acetone were received from Aladdin Co., Ltd. Polytetrafluoroethylene (PTFE) and sodium dodecyl benzene sulfonate were purchased from the Huangjiang Co., Ltd. (Dongguan, China). ER and curing agent were purchased from Sanmu Co. Ltd. (Suzhou, China). Deionized water (resistivity 18 MΩcm) was utilized to prepare all aqueous solutions.

### Preparation

The preparation of the RGO nanosheets and 3DGNs has been reported by our group [12–14], and more details are provided in the Supplementary materials. The RGO-3DGNs-ER composite was fabricated by a two-step method. Firstly, the combination of the RGO nanosheets and 3DGNs is achieved by a simple hydrothermal method. A certain amount of the RGO nanosheets and 3DGNs was added into 50 ml deionized water, and a 30-min ultrasonic process is carried out. After that, 1 mg sodium dodecyl benzene sulfonate was added, and then the mixture was moved into a Teflon vessel for hydrothermal reaction at 80 °C for 6 h. Then, the resulting material was washed with deionized water for three times, and the RGO nanosheets were loaded on the surface of the 3DGNs. Secondly, the preparation of the RGO-3DGNs-ER is similar with our reported 3DGNs-ER [7]. Briefly, a certain amount of prepared RGO-3DGNs was put into a mold, and the ER including the curing agent was dropped on the solid surface. After dropping a layer of the ER, the RGO-3DGNs was added again. The two steps are repeated for three or four times. The dropped ER penetrates into the porous RGO-3DGNs by capillary effect. Finally, the RGO-3DGNs-ER mixture was cured at 110 °C for 3 h.

### Characterization

Morphology of the TIMs was obtained by a scanning electron microscope (SEM, FEI Sirion 200 scanning electron microscope working at 5 kV) and transmission electron microscope (TEM, JEM-2100F, operated at an accelerating voltage of 20 kV). Atomic force microscopy (AFM) results were recorded by Nanoscope IIIa (Digital Instrument, USA) and E-Sweep (Seiko, Japan) in tapping mode. Scanning Raman spectra were recorded by LabRam-1B Raman microspectrometer at 532 nm (Horiba Jobin Yvon, France). X-ray photoelectron spectroscopy (XPS) measurements were performed on a RBD upgraded PHI-5000C ESCA system (Perkin Elmer). Fourier transform infrared spectroscopy (FTIR) curves were measured on IR Prestige-21 system (PerkinElmer). Mechanical properties of these composites were recorded by a Triton DMTA (Triton Instrument, UK) instrument. The Tg and storage modulus were measured at a frequency of 1 Hz and a heating rate of 5 °C min^−1^ according to ASTM1640 and analyzed in the tensile mode. The dimensions of the samples were 2 × 4 cm. Laser flash analysis and differential scanning calorimetry were used to analyze the thermal transport performance of the fabricated composites.

## Results and Discussion

**Fig. 1:**
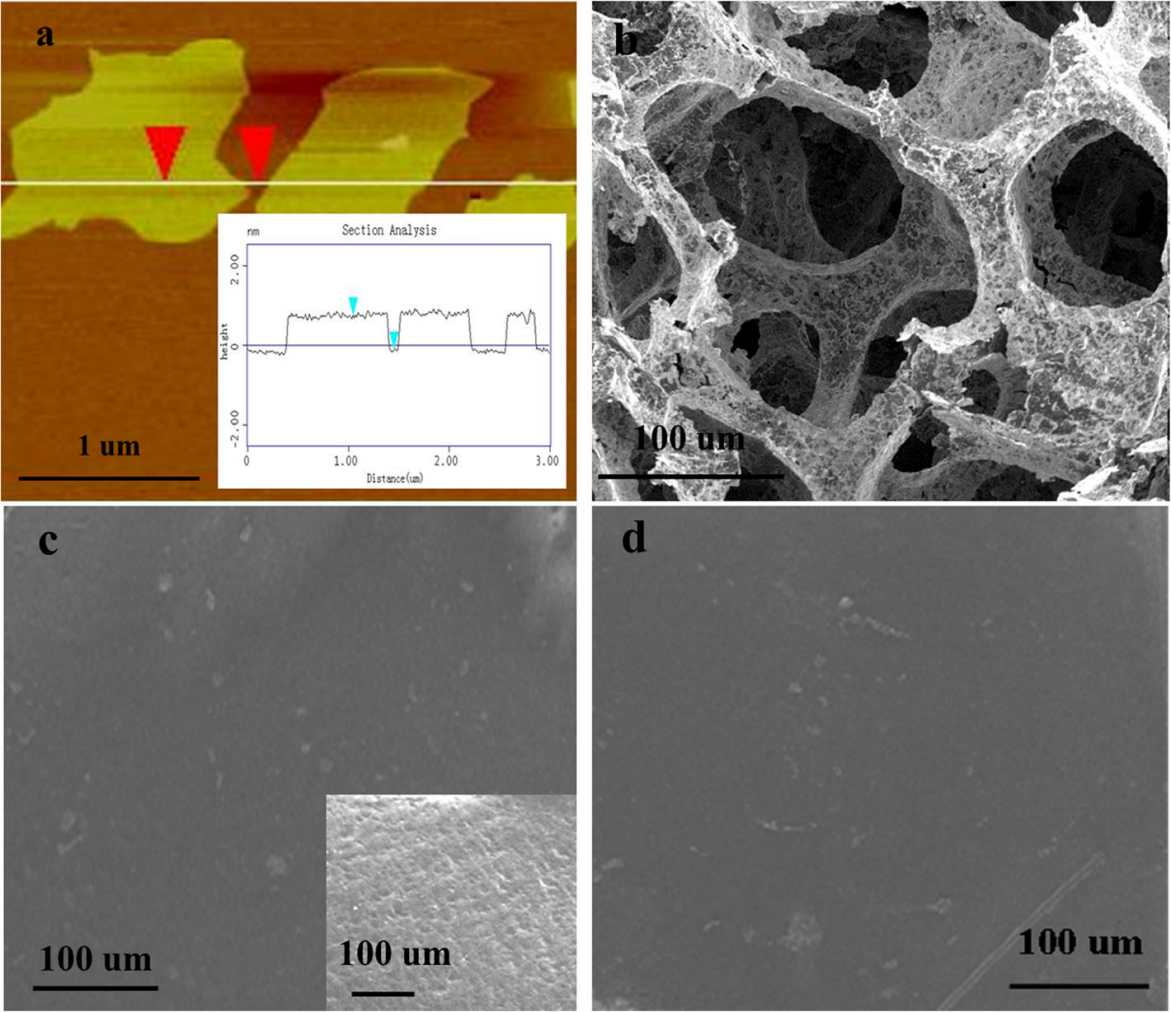
Morphologies of the RGO nanosheets, 3DGNs, and resulting TIMs. AFM and SEM images of the prepared RGO nanosheets, 3DGNs, RGO-3DGNs, and RGO-3DGNs-ER are shown in Fig. 1. The average size of the RGO nanosheets is 400~600 nm **a**, which is elaborately designed to combine with the 3DGNs by adjusting the oxidation and reduction procedures. A continuous 3D construction of the 3DGNs can be seen from **b**, and its porous structure is clearly shown. As for the resulting TIM, the smooth surface of the RGO-ER can be seen from **c**, and the absence of tiny pores (compared with that of the pristine ER, inset of **c** indicates a potential high thermal performance. **d** The morphology of the RGO-3DGNs-ER, which is similar with that of the RGO-ER. The 3D structure of the 3DGNs is difficult to identify in the SEM image because the 3D interspaces are filled by the ER. However, the 3D phonon transport network (the function of the 3DGNs) still maintains in the TIMs, which has been proven by our previous reports. The RGO nanosheets in the RGO-3DGNs-ER should be loaded on the surface of the 3DGNs because of the hydrothermal reaction, which is the pre-condition to exert the function (enhance the wettability between the graphene basal plane and ER) of the RGO nanosheets

AFM and SEM images of the prepared RGO nanosheets, 3DGNs, RGO-3DGNs, and RGO-3DGNs-ER are shown in Fig. 1. The average size of the RGO nanosheets is 400~600 nm (Fig. 1a), which is elaborately designed to combine with the 3DGNs by adjusting the oxidation and reduction procedures. A continuous 3D construction of the 3DGNs can be seen from Fig. 1b, and its porous structure is shown clearly. As for the resulting TIM, the smooth surface of the RGO-ER can be seen from Fig. 1c, and the absence of tiny pores (compared with that of the pristine ER, inset of Fig. 1c) indicates a potential high thermal performance. Figure 1d displays the morphology of the RGO-3DGNs-ER, which is similar with that of the RGO-ER. The 3D structure of the 3DGNs is difficult to identify in the SEM image because the 3D interspaces are filled by the ER. However, the 3D phonon transport network (the function of the 3DGNs) still maintains in the TIMs, which has been proven by our previous reports [7]. The RGO nanosheets in the RGO-3DGNs-ER should be loaded on the surface of the 3DGNs because of the hydrothermal reaction, which is the pre-condition to exert the function (enhance the wettability between the graphene basal plane and ER) of the RGO nanosheets (more details will be discussed in the following).

**Fig. 2:**
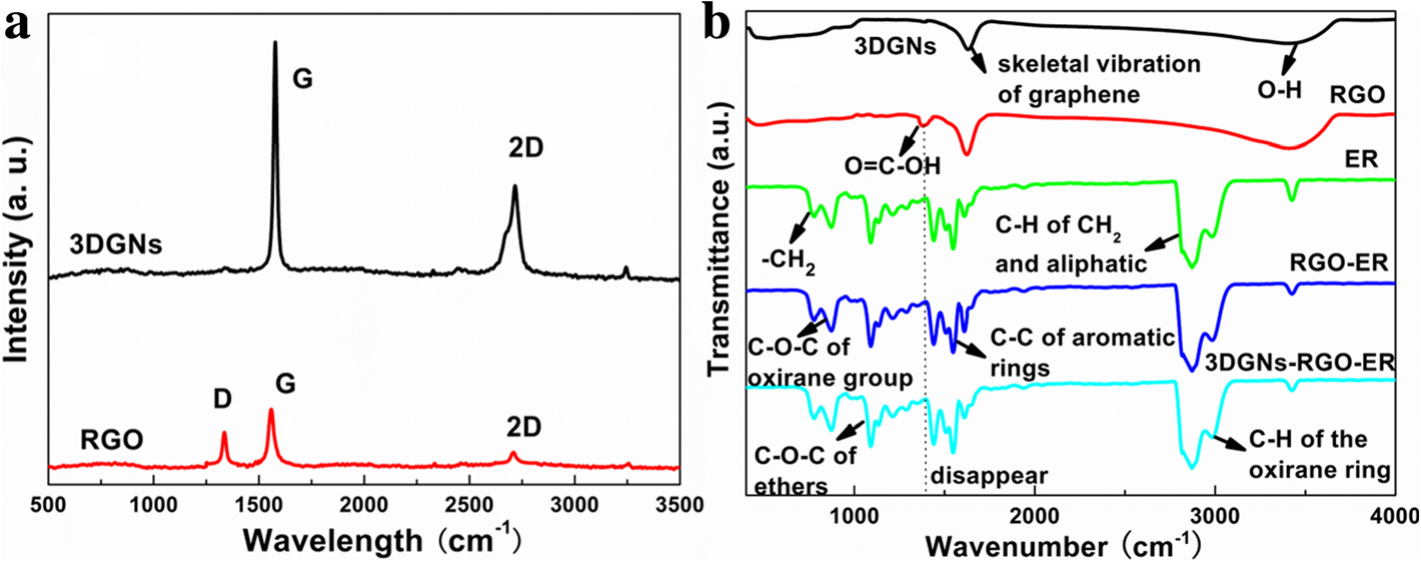
Raman and FTIR curves of the various samples. Raman curves of the adopted RGO nanosheets and 3DGNs are shown in **a**. Three major signals, G, 2D, and D peaks, can be seen for the former, while the D peak is difficult to find in the corresponding pattern of the 3DGNs. As for the graphite-like materials, the D peak is aroused from defects. Therefore, the obtained Raman profile implies the high quality of the 3DGNs. The G band associates with the E_2g_ phonon at Brillouin zone center. Moreover, the defect density and average size of the RGO nanosheets can be calculated by the integrated intensity ratio of *I*_G_/*I*_D_. After calculation, the average size is ~ 500 nm, which is in line with the result of the SEM image. The enlarged FTIR is a useful tool to observe the chemical bond between various materials according to the intensities and positions of the corresponding signals. The major adsorption peaks and the corresponding functional groups of the ER are marked in **b**, and the spectra of the RGO nanosheets and 3DGNs are also presented. The similar signals at ~ 1600 cm^−1^ and 3000–3700 cm^−1^ are induced from the skeletal vibration of the graphene basal plane and the O–H stretching vibration of adsorbed water. A remarkable difference between these two profiles is that an additional obvious peak at 1335 cm^−1^ arising from the O=C–OH can be seen only for the RGO nanosheets resulting from the surface functional groups. After combining with the ER, the O=C–OH signal disappears absolutely, manifesting that the carboxyl on the surface of the RGO nanosheets reacts with hydroxyl of the ER to form a close chemical contact, which contribute to the phonon fast transport at the interface between them

Raman curves of the adopted RGO nanosheets and 3DGNs are shown in Fig. 2a. Three major signals, G, 2D, and D peaks, can be seen for the former, while the D peak is difficult to find in the corresponding pattern of the 3DGNs. As for the graphite-like materials, the D peak is aroused from defects. Therefore, the obtained Raman profile implies the high quality of the 3DGNs [15, 16]. The G band associates with the E_2g_ phonon at Brillouin zone center. Moreover, the defect density and average size of the RGO nanosheets can be calculated by the integrated intensity ratio of *I*_G_/*I*_D_ [15]. According to Eq. (1) [17],

1 $$ {L}_a=\frac{43.5}{R}=43.5\times \frac{I_G}{I_D} $$

the average size is ~ 500 nm, which is in line with the result of the AFM image. Two kinds of defects including functional groups and boundaries can be classified for the RGO nanosheets. The amount of boundaries is determined by the average size of the adopted RGO nanosheets, while the amount of the functional group is dependent on the reduction procedure. More details on the reduction degree of the RGO nanosheets by XPS spectra are discussed in our previous reports and the Supplementary materials [7, 8]. The enlarged FTIR is a useful tool to observe the chemical bond between various materials according to the intensities and positions of the corresponding signals. The major adsorption peaks and the corresponding functional groups of the ER are marked in Fig. 2b, and the spectra of the RGO nanosheets and 3DGNs are also presented. The similar signals at ~ 1600 cm^−1^ and 3000–3700 cm^−1^ are induced from the skeletal vibration of the graphene basal plane and the O–H stretching vibration of adsorbed water [18–20]. A remarkable difference between these two profiles is that an additional obvious peak at 1335 cm^−1^ arising from the O=C–OH can be seen only for the RGO nanosheets resulting from the surface functional groups [21]. After combining with the ER, the O=C–OH signal disappears absolutely, manifesting that the carboxyl on the surface of the RGO nanosheets reacts with hydroxyl of the ER to form a close chemical contact, which contributes to the phonon fast transport at the interface between them.

**Fig. 3:**
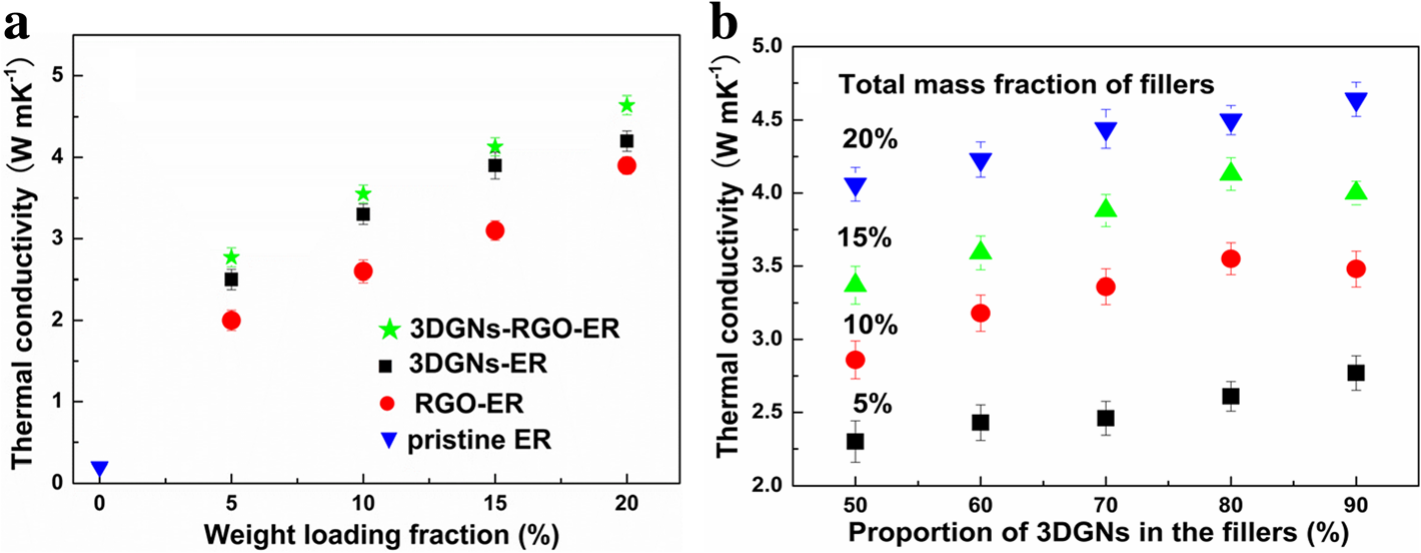
Thermal conductivities of resulting composites with increased mass factions of fillers. The corresponding thermal performances of various samples are shown in Fig. 3. The thermal conductivity of the pristine ER is 0.2 W/mK, which is far from the requirement for the TIMs. With the increased mass fractions of various fillers, the resulting thermal performances enhance almost in a linearly manner (**a**). Therein, the RGO nanosheets and 3DGNs co-modified composites display the best performance with identical mass fraction compared with these cases of employing a single filler, and the specific thermal conductivity value is closely related to the proportion of the 3DGNs and RGO nanosheets, demonstrating a synergy between them (**b**). Although both the RGO nanosheets and 3DGNs are constituted with graphene basal sheets, the distinctions from morphology of these two fillers and chemical state of carbon atoms endow the different functions of them in the TIMs. On the one hand, the high quality and the continuous structure of the 3DGNs make it an excellent fast transport network for phonons, which has been proven in our previous reports. On the other hand, due to the high defect density and the lack of a continuous structure, the phonon transport ability of the RGO filler is weaker than the 3DGNs

**Fig. 4:**
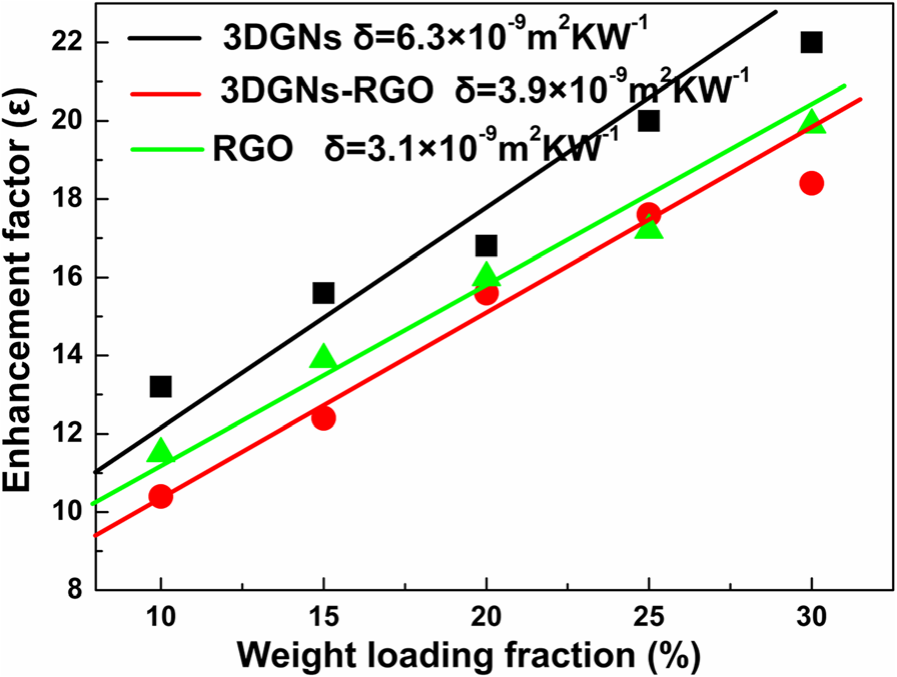
Calculated thermal boundary resistance of the various samples. Thermal boundary resistance (δ) is an important parameter to determine the resulting thermal performances of TIMs. Based on Balandin’s theory, thermal conductivity of graphene-modified ER is closely related to the value of the δ. After calculation, the similar δ values of the RGO-ER and RGO-3DGNs-ER samples prove that the added RGO nanosheets are loaded on the surface of the 3DGNs (Fig. 4). Based on our previous findings, the δ value of the 3DGNs-ER sample is much higher that of the RGO-ER because of the poor contact between the 3DGNs and ER. The functional groups of the RGO nanosheets bring about a better contact at the interface, which leads to the smaller δ compared with that of 3DGNs-ER sample

The corresponding thermal performances of various samples are shown in Fig. 3. The thermal conductivity of the pristine ER is ~ 0.2 W/mK, which is far from the requirement for the TIMs in the practical application. With the increased mass fractions of various fillers, the resulting thermal performances enhance almost in a linear manner (Fig. 3a). Therein, the RGO nanosheets and 3DGNs co-modified composites display the best performance with identical mass fraction compared with these cases of employing a single filler, and the specific thermal conductivity value is closely related to the proportion of the 3DGNs and RGO nanosheets, demonstrating a synergy between them (Fig. 3b). Although both the RGO nanosheets and 3DGNs are constituted with graphene basal sheets, the distinctions from the morphology of these two fillers and chemical state of carbon atoms endow the different functions of them in the TIMs. On the one hand, the high quality and the continuous structure of the 3DGNs make it an excellent fast transport network for phonons, which has been proven in our previous reports [8]. On the other hand, due to the high defect density and the lack of a continuous structure, the phonon transport ability of the RGO filler is weaker than the 3DGNs [7]. Therefore, the general performances of the RGO nanosheet-assisted TIMs are not as good as these samples of adopting the 3DGNs. However, surface functional groups of the RGO nanosheets bring about a better contact for the interface between the graphene basal plane and ER, which can be confirmed by the reduced thermal boundary resistance. Based on Balandin’s theory, thermal conductivity of graphene-modified ER can be expressed as follows [22]:

2 $$ K={K}_g\left[\frac{2p\left({K}_g-{K}_e\right)+3{K}_e}{\left(3-p\right){K}_g+{K}_ep+\frac{\delta {K}_g{K}_ep}{H}}\right] $$

where *p* represents the volume percentage of the graphene filler and *K*, *K*_*g*_, and *K*_*e*_ are thermal conductivities of the resulting composite, graphene, and ER, respectively. *H* and *δ* are the thickness of the graphene and the thermal boundary resistance between the graphene and ER. After calculation, the similar *δ* values of the RGO-ER and RGO-3DGNs-ER samples prove that the added RGO nanosheets are loaded on the surface of the 3DGNs (Fig. 4). Based on our previous findings, the *δ* value of the 3DGNs-ER sample is much higher than that of the RGO-ER because of the poor contact between the 3DGNs and ER [7, 8]. The functional groups of the RGO nanosheets bring about a better contact at the interface, which leads to the smaller *δ* compared with that of 3DGNs-ER sample. The further optimization on the reduction degree of the adopted RGO nanosheets is carried out, and the ratio of the element carbon atoms to carbon atoms from functional groups ~ 1.7 is recommended (more details are provided in Additional file 1: Figure S1 and our previous reports [7, 8]).

**Fig. 5:**
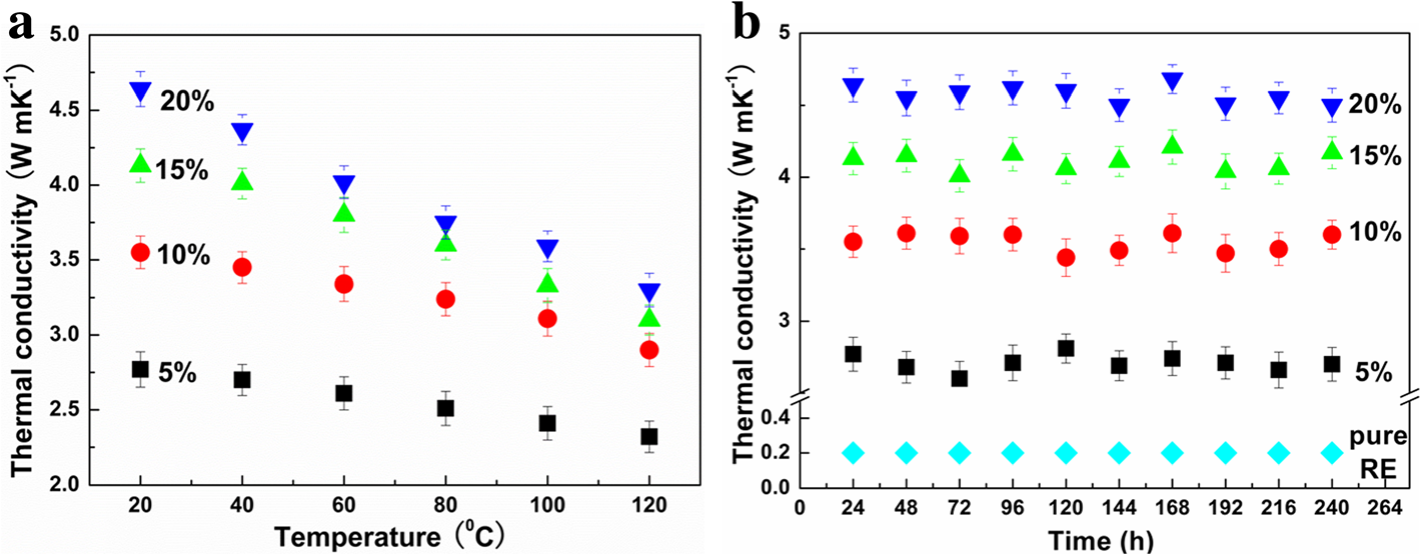
Calculated thermal boundary resistance of the various samples. In order to simulate the practical work condition of electronic devices, the performances of the resulting TIMs under high temperature are detected (**a**). With increased temperature, the thermal conductivities of all TIMs decrease due to the enhanced Umklapp scattering. Although the Kapitza boundary scattering decreases at the same time (the probability of a phonon across the interface is proportional to $\sim {e}^{\frac{-E}{KT}}$), the decrease cannot remedy the corresponding increase of the Umklapp scattering, leading to the whole decrease of thermal conductivity. Compared with that of the 3DGN-assisted sample, the stability of thermal conductivity of the RGO nanosheets added composites under high temperature is better because of the more sensitive Kapitza boundary scattering (as a results of the more boundaries of the RGO nanosheets). Moreover, no obvious degradation can be found for the thermal performance of the RGO-3DGNs-ER sample after 240 h continuous working (**b**), indicating the potential promising prospect of this TIM. The stability of the pure ER during a long work time is also recorded in **b**. The similar stabilities of the pure ER and the resulting composites (all the degradations of their thermal conductivities are less than 10%) indicate that no significant influence on the thermal stability can be found after adding the fillers

In order to simulate the practical work condition of electronic devices, the performances of the resulting TIMs under high temperature are detected (Fig. 5a). With increased temperature, the thermal conductivities of all TIMs decrease due to the enhanced Umklapp scattering. Although the Kapitza boundary scattering decreases at the same time (the probability of a phonon across the interface is proportional to $\sim {e}^{\frac{-E}{KT}}$), the decrease cannot remedy the corresponding increase of the Umklapp scattering, leading to the whole decrease of thermal conductivity. Compared with that of the 3DGN-assisted sample, the stability of thermal conductivity of the RGO nanosheets added composites under high temperature is better because of the more sensitive Kapitza boundary scattering (as a result of the more boundaries of the RGO nanosheets). Moreover, no obvious degradation can be found for the thermal performance of the RGO-3DGNs-ER sample after 240 h continuous working (Fig. 5b), indicating the potential promising prospect of this TIM. The stability of the pure ER during a long work time is also recorded in Fig. 5b. The similar stabilities of the pure ER and the resulting composites (all the degradations of their thermal conductivities are less than 10%) indicate that no significant influence on the thermal stability can be found after adding the fillers.

As last, the mechanical properties of these TIMs are also recorded. The corresponding performances including ultimate strengths and stretching limits of them are listed in Additional file 1: Table S1. Both the 3DGNs-ER and RGO-3DGNs-ER samples display the high mechanical strength because the continuous 3D structure of the 3DGNs is beneficial to keeping the outstanding intrinsic mechanical property of the graphene. After comparing the performances of the 3DGNs-ER and RGO-3DGNs-ER samples, it can be inferred again that the RGO nanosheets are loaded on the surface of the 3DGNs rather than dispersed in the ER matrix because the influence from the added RGO nanosheets can be ignored.

## Conclusions

The RGO nanosheets and 3DGNs co-modified ER has been prepared to prepare the TIMs. The advantages of the RGO nanosheets and 3DGNs can give full play to loading the RGO nanosheets on the surface of 3DGNs (by a hydrothermal process) rather than dispersing in the ER matrix. The presence of the 3DGNs not only provides a fast transport network for phonons but also acts as a scaffold for the RGO nanosheets. On the other hand, the surface functional groups of the RGO nanosheets enhance the close contact between the graphene basal plane and ER at their interface, which offsets the poor wettability of the 3DGNs. Therefore, the thermal performance of the resulting TIM is enhanced significantly (a high thermal conductivity ~ 4.6 W/mK is achieved when a 9 wt% 3DGNs and 1 wt% RGO nanosheets are added, which is 10 and 36% higher than those cases of 10 wt% 3DGNs and 10 wt% RGO nanosheet added samples), and a well stability of the thermal performance of the resulting TIM is revealed under high temperature (at 100 °C, the decrease of the thermal conductivity is less than 25%). Moreover, the excellent mechanical properties including high ultimate strength and stretch limits indicate the potential promising prospect of the presented TIM. 

## Additional file

Additional file 1: Figure S1. XPS curve of RGO after reduction with optimizing time. **Table S1.** Mechanical performances of the various samples (DOCX 186 kb)

## References

[1] Gallego MM, Verdejo R, Khayet M (2011). Nanoscale Res Lett.

[2] Jung DY, Yang SY, Park H (2015). Nanoscale Res Lett.

[3] Fu YX, He ZX, Mo DC (2014). Appl Therm Eng.

[4] Im H, Kim J (2012). Carbon.

[5] Xie QZ, Zhu QZ, Li Y (2016). Nanoscale Res Lett.

[6] Ghosh S, Calizo I, Teweldebrhan D (2008). Appl Phys Lett.

[7] Tang B, Hu GX, Gao HY (2015). Int J Heat Mass Transf.

[8] Sun YF, Tang B, Huang WQ (2016). Appl Therm Eng.

[9] Wei JC, Vo T, Inam F (2015). RSC Adv.

[10] Tang B, Wang SL, Zhang J et al (2017) Int Mater Rev 10.1080/09506608.2017.1344377

[11] Zhang J, Lin S, Tang B et al (2017) Nanoscale Res Lett. 10.1186/s11671-017-2224-4

[12] Tang B, Hu GX (2014). Chem Vapor Depos.

[13] Gao HY, Xue C, Hu GX (2017). Ultrason Sonochem.

[14] Gao HY, Zhu KX, Hu GX (2016). Chem Eng J.

[15] Tang B, Hu GX, Gao HY (2010). Appl Spectrosc Rev.

[16] Ferrari AC, Meyer JC, Scardaci V (2006). Phys Rev Lett.

[17] Gupta A, Chen G, Eklund PC (2006). Nano Lett.

[18] Zhang H, Lv XJ, Li YM (2010). ACS Nano.

[19] Neumann B, Bogdanoff P, Tributsch H (2005). J Phys Chem B.

[20] Xiao Q, Zhang J, Xiao C (2008). Sol Energy.

[21] Xiong WL, Chen Y, Hao M (2015). Appl Therm Eng.

[22] Khan MF, Balandin AA (2012). Nano Lett.

