# Supplementary material for: RGO and Three-Dimensional Graphene Networks Co-modified TIMs with High Performances
Source: Nanoscale Res Lett. 2017 Sep 6;12:527. doi: 10.1186/s11671-017-2298-z (PMC5585116; doi:10.1186/s11671-017-2298-z)
Supplement: Additional file 1: Figure S1. — XPS curve of RGO after reduction with optimizing time. Table S1. Mechanical performances of the various samples (DOCX 186 kb) [file 11671_2017_2298_MOESM1_ESM.docx]

**RGO and three-dimensional graphene networks co-modified TIMs with high-performance**

Bo Tang*, Zhengwei, Wang, Weiqiu Huang*, Sen Li, Tingting Ma, Haogang, Yu, Xufei, Li

School of Petroleum Engineering, Changzhou University, Changzhou city 213016, China

Corresponding author: Tel./fax: +86 519 83295530. E-mail: [tangbo@cczu.edu.cn](mailto:tangbo@cczu.edu.cn); hwq213@cczu.edu.cn

**Preparation of the 3DGNss and RGO**

An additional unit in the chemical vapor deposition equipment was adopted to put the nickel foam rod (NFR), which is important to achieve the concentration gradients of the CH_4_ gas as well as a controllable thickness of the 3DGNs. The NFR was pre-treated by diluted acetic acid and deionized water in ultrasonic processing to remove the oxidation and dust. Then the NFR was heated to 1100 °C under the Ar (300 standard cubic centimeter per minute, sccm) and H_2_ (150 sccm) conditions with a 20 °C min^-1^ rate. A small amount of CH_4_ (10 sccm) was introduced into the tube furnace when the required temperature was achieved. After 10 min, the samples were cooled to room temperature under the Ar (300 sccm) and H_2_ (100 sccm) conditions. In order to obtain the freestanding 3DGNs, the NFR was removed by HCl (1 mol L^-1^) solution at 80 °C. A thin poly methylmethacrylate (PMMA) was used as a support material (dip-coating) to protect the foam-like structure of the 3DGNs. Finally, the free-standing 3DGNs was obtained by dissolving the PMMA with acetone solution at 60 °C.

Modified Hummers’ method was used to prepare the graphene oxide (GO), and then the hydrazine was adopted to achieve the reduction of the GO to the reduced graphene oxide (RGO) in 98^0^C water bath for 2 h.


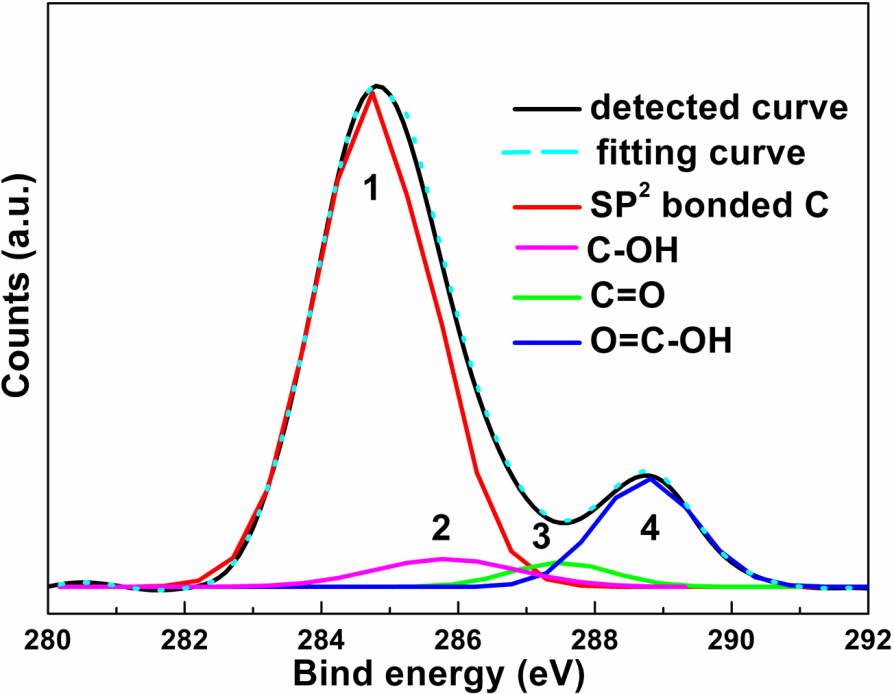


Fig. S1 XPS curve of RGO after reduction with optimizing time.

Table S1 Mechanical performances of the various samples.

| Property  Samples | Ultimate strength  (enhancement factor) | Stretch limits  (strain %) |
| --- | --- | --- |
| Pristine ER | 1  0.99  0.97  0.92  1.04  1.26  1.17  1.05  1.10  1.12  1.09  1.16  1.21  0.98  1.01  1.05 | 230%  230%  220%  210%  260%  280%  280%  230%  240%  240%  230%  250%  270%  240%  260%  260% |
| RGO(5%)-ER |  |  |
| RGO(10%)-ER |  |  |
| RGO(15%)-ER |  |  |
| 3DGNss(5%)-ER |  |  |
| 3DGNss(10%)-ER |  |  |
| 3DGNss(15%)-ER |  |  |
| RGO(2.5%)-3DGNss(2.5%)-ER |  |  |
| RGO(1.5%)-3DGNss(3.5%)-ER |  |  |
| RGO(0.5%)-3DGNss(4.5%)-ER |  |  |
| RGO(5%)-3DGNss(5%)-ER |  |  |
| RGO(2%)-3DGNss(8%)-ER |  |  |
| RGO(1%)-3DGNss(9%)-ER |  |  |
| RGO(7.5%)-3DGNss(7.5%)-ER |  |  |
| RGO(6%)-3DGNss(9%)-ER |  |  |
| RGO(3%)-3DGNss(12%)-ER |  |  |
